# Interleukin-1 Beta Neutralization Attenuates Traumatic Brain Injury-Induced Microglia Activation and Neuronal Changes in the Globus Pallidus

**DOI:** 10.3390/ijms21020387

**Published:** 2020-01-08

**Authors:** Ilknur Ozen, Karsten Ruscher, Robert Nilsson, Johanna Flygt, Fredrik Clausen, Niklas Marklund

**Affiliations:** 1 Lund Brain Injury Laboratory for Neurosurgical Research, Department of Clinical Sciences, Lund University, 22184 Lund, Sweden; ilknur.ozen@med.lu.se (I.O.); karsten.ruscher@med.lu.se (K.R.); nilsson.f.robert@gmail.com (R.N.); 2 Laboratory for Experimental Brain Research, Department of Clinical Sciences, Lund University, 22184 Lund, Sweden; 3 Department of Neuroscience, Section of Neurosurgery, Uppsala University, 75185 Uppsala, Sweden; johanna.flygt@ctc-ab.se (J.F.); fredrik.clausen@neuro.uu.se (F.C.); 4 Department of Clinical Sciences Lund, Neurosurgery, Lund University, 22185 Lund, Sweden

**Keywords:** traumatic brain injury, interleukin-1β, inflammation, Parkinson’s disease, globus pallidus, axonal injury

## Abstract

Traumatic brain injury (TBI) increases the risk of delayed neurodegenerative processes, including Parkinson’s disease (PD). Interleukin-1beta (IL-1β), a key pro-inflammatory cytokine, may promote secondary injury development after TBI. Conversely, neutralizing IL-1β was found to improve functional recovery following experimental TBI. However, the mechanisms underlying the behavioral improvements observed by IL-1β neutralization are still poorly understood. The present study investigated the role of IL-1β on the microglia response and neuronal changes in the globus pallidus in response to diffuse TBI. Mice were subjected to sham injury or the central fluid percussion injury (cFPI) (a model of traumatic axonal injury), and were randomly administered an IL-1β neutralizing or a control antibody at 30 min post-injury. The animals were analyzed at 2, 7, or 14 days post-injury. When compared to controls, mice subjected to cFPI TBI had increased microglia activation and dopaminergic innervation in the globus pallidus, and a decreased number of parvalbumin (PV) positive interneurons in the globus pallidus. Neutralization of IL-1β attenuated the microglia activation, prevented the loss of PV+ interneurons and normalized dopaminergic fiber density in the globus pallidus of brain-injured animals. These findings argue for an important role for neuro-inflammation in the PD-like pathology observed in TBI.

## 1. Introduction

Traumatic brain injury (TBI) is a common cause of disability and death, particularly in patients < 40 years of age [1]. In TBI, the initial physical impact results in a primary brain injury that is followed by a secondary disease process. Key contributors to the secondary injury process are microglia cell activation and elevated pro-inflammatory cytokines that persist for many years post-injury in both experimental and human TBI [2,3].

The cytokine Interleukin-1β (IL-1β) is one of the most important pro-inflammatory cytokines, increased early following experimental and human TBI, and mainly secreted by activated microglia, but also by damaged endothelial cells, and astrocytes [4,5]. Strong expression of IL-1β together with other pro-inflammatory cytokines such as IL-6, IL-4 and interferon-gamma occurs in the perilesional zone in both the early and delayed phase after TBI in humans [6]. Importantly, the production of IL-1β is closely associated with injury severity. Both moderate and severe TBI increase IL-1β mRNA and protein levels in the cortex and deep central brain structures as early as 1 h after the injury [7]. IL-1β has a role in glutamate excitotoxicity, and may stimulate production of cytokines and prostaglandins [8,9]. Thereby, IL-1β can be a promising therapeutic target for TBI. Indeed, we previously showed that systemic administration of a neutralizing IL-1β antibody attenuated the inflammatory response, cerebral edema, and tissue loss and improved cognitive outcome in a focal TBI model in mice. Furthermore, neutralization of IL-1β attenuated the complex behavioral and cognitive dysfunction observed in mice subjected to the central fluid percussion model [10]. Similarly, systemic post-injury administration of a human recombinant interleukin-1 receptor antagonist resulted in attenuated neuronal loss and oligodendrocyte death after lateral fluid-percussion brain injury [11,12]. We have also shown that the number of microglia cells are increased and that they undergo morphological changes in white matter after diffuse TBI, changes normalized by IL-1β neutralization [12]. However, the effects of IL-1β neutralization on TBI-induced microglia response in midbrain regions have not been established.

TBI is an established risk factor for neurodegenerative diseases later in adult life, including Parkinson’s disease (PD) [13,14,15,16]. TBI may induce PD-like symptoms in humans and in animal models [16,17,18,19,20,21]. For instance, α-synuclein (α-syn) levels were increased in cerebrospinal fluid in clinical TBI and in the substantia nigra (SN) in animal TBI models. This PD-like pathology observed in TBI may plausibly be related to degeneration of dopamine-producing neurons and dysfunction of the basal ganglia [22,23].

The globus pallidus (GP) is part of an indirect pathway of the basal ganglia circuitry that connects the striatum with the subthalamic nucleus, and GP is one of the most important deep central brain structures [24]. The GP is consistent of network of GABAergic neurons and plays a key role in Parkinson’s disease (PD) pathology. In PD, hypoactivity of the GP may cause increased thalamic and cortical activity that, in return leads to akinesia [25,26,27]. The GP has been shown to be highly susceptible to injury following focal TBI in humans. However, how TBI results in PD-related pathology including GP dysfunction has not been evaluated.

In this study, we examined the microglia response and neuronal changes in the GP in a mouse model of diffuse TBI, the central fluid percussion model of widespread traumatic axonal injury, and investigated the effects of IL-1β neutralization on (i) microglial cells, (ii) parvalbumin (PV) positive interneurons, and (iii) dopaminergic innervation of the GP. We showed for the first time that the central fluid percussion injury (cFPI) model in mice results in microglia activation and a significant decrease in the number of PV+ interneurons, as well as increased tyrosine hydroxylase (TH) positive axon density in the GP. Neutralizing antibody IL-1β treatment inhibited the microglia response, attenuated the loss of PV+ interneurons and normalized the TH positive axon density in the GP of brain-injured animals.

## 2. Results

### 2.1. Neutralization of IL-1beta Attenuates TBI-Induced Microglia Activation in the Globus Pallidus

Iba1 has been shown to be highly and specifically expressed in endogenous microglia in rodent brain [28]. Most importantly, Iba1 protein is strongly upregulated in activated microglia, which makes it a good marker for detecting the activation of microglia following TBI [29]. In previous work from our group, we found that Iba1 was a robust marker of microglia activation in the cFPI model [12].

**Figure 1:**
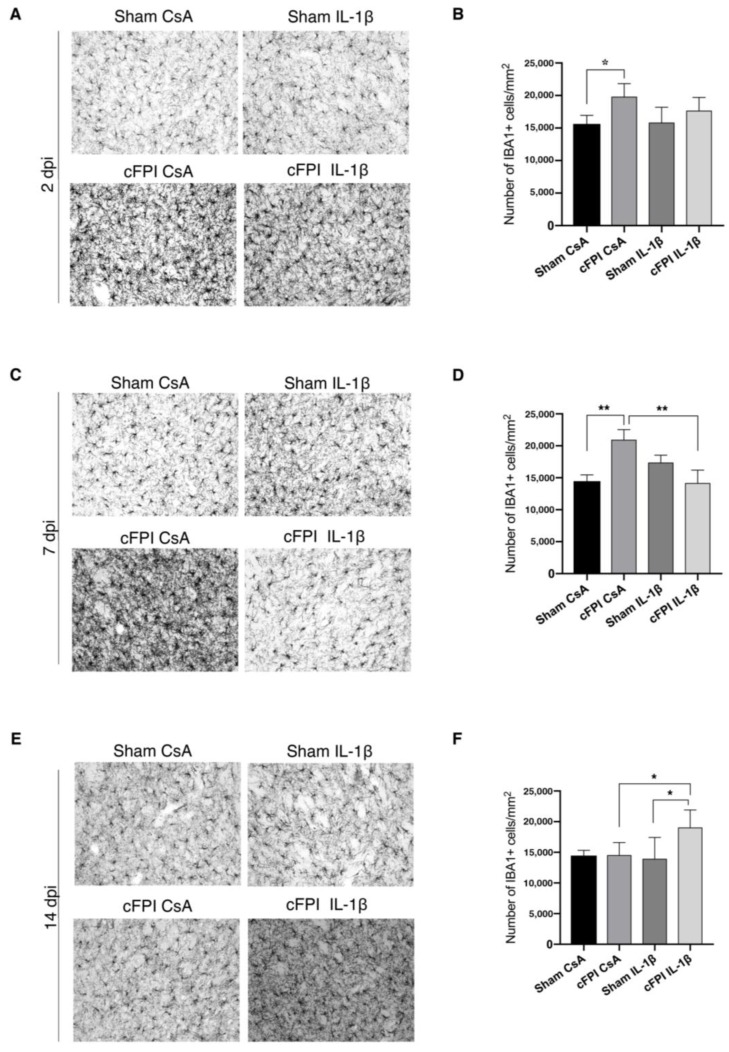
IL-1β neutralization attenuates the increase of Iba1+ microglia cells in the globus pallidus. Compared with sham CsA mice at two dpi, (**A**,**B**) the number of Iba1+ cells was increased in CsA cFPI animals (sham CsA, *n* = 4; sham IL-1β, *n* = 3; cFPI CsA, *n* = 6; cFPI IL-1β, *n* = 6; * *p* ≤ 0.05), while neutralizing IL-1 β had no effect on the number of Iba1+ microglial cells. At two dpi, Iba1 positive microglia processes were thin and ramified in the sham CsA group compared with thicker processes in the cFPI CsA animals (**C**,**D**) At seven dpi, the increase in the number of Iba1+ microglia cells persisted in the cFPI CsA animals compared with sham groups, which was significantly reduced by IL-1β neutralizing antibody treatment (sham CsA, *n* = 4 sham IL-1β, *n* = 3; cFPI CsA, *n* = 7; cFPI IL-1β, *n* = 6; ** *p* ≤ 0.01). Iba-1 positive microglia had thin and ramified processes in the cFPI IL-1β animals similar to sham CsA group (**E**,**F**) At 14 dpi, no difference was observed in the number of Iba1 positive microglia cells in the cFPI CsA animals in comparison to the sham groups (sham CsA and sham IL-1β). In the GP, the Iba-1 immunoreactivity was significantly increased by IL-1β neutralization in the injured mice (sham CsA, *n* = 3; sham IL-1β, *n* = 3; cFPI CsA, *n* = 8; cFPI IL-1β, *n* = 11; * *p* ≤ 0.05). Iba1 positive microglia cells had thicker processes in the cFPI IL-1β animals as compared to other groups. cFPI, central fluid percussion injury; dpi, days post-injury; CsA, inactive control antibody against cyclosporin A; IL-1β, interleukin 1 beta; Scale bars 20 μm.

In brain-injured animals subjected to cFPI (cFPI CsA), a significant increase in the number of Iba1+ microglia cells was observed in the globus pallidus (GP) starting at two days post-injury (dpi, Figure 1A,B) as compared to sham-injured, control antibody-treated animals (Sham CsA). This increase in Iba1+ microglia cells was maintained up to seven dpi, however then decreased to levels similar to Sham CsA group at 14 dpi (Figure 1C–F). Iba1+ microglia cells in the GP of the sham-injured animals had resting morphology characterized by a small cell body with highly ramified processes. Importantly, the number of the microglia cells remained unchanged at 2, 7, and 14 dpi (Figure 1A,C,F). However, Iba1+ microglia cells had morphological changes consistent with microglia activation, including less ramified and thicker processes, and swollen cell bodies at 2, 7, and 14 dpi in the GP of the cFPI animals (Figure 1A,C,F). We then analyzed whether IL-1β neutralization influenced the number of Iba1+ microglia cells in the GP (Figure 1). At two dpi, the number of Iba1+ cells was similar in the cFPI CsA and the cFPI IL-1β groups (Figure 1A,B). At seven dpi, the number of Iba1+ cells in the cFPI IL-1β group was significantly lower when compared to the cFPI CsA group, and neutralizing IL-1β treatment normalized the number of Iba1+ cells to levels similar to the sham CsA group (Figure 1C,D). At 14 dpi, the number of Iba1+ microglia cells was similar in the cFPI CsA as compared to the sham CsA group, whereas IL-1β neutralized brain-injured animals had a significantly increased number of Iba1+ microglia cells at when compared to the cFPI CsA and sham IL-1β groups (Figure 1E,F).

### 2.2. IL-1beta Neutralization Prevents Loss of Parvalbumin Positive Interneurons in the Globus Pallidus Following TBI

**Figure 2:**
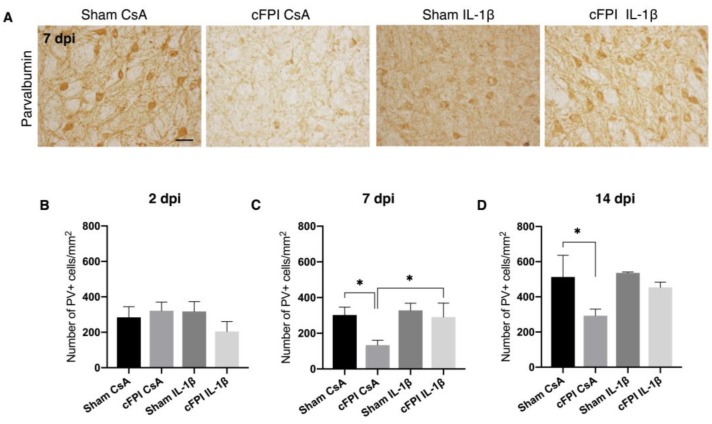
Neutralizing IL-1β antibody rescues Parvalbumin-positive neurons in the globus pallidus (**A**) Representative images showing PV+neurons in the GP at seven dpi, scale bar 20 μm. (**B**) Compared with sham-injured, control-treated animals (Sham CsA) at two dpi, there was no change in the number of PV+ neurons (sham CsA, *n* = 4; sham IL-1β, *n* = 3; cFPI CsA, *n* = 6; cFPI IL-1β, *n* = 6) (**C**) At seven dpi, the number of PV+ neurons decreased significantly in the brain-injured, control-treated (cFPI CsA) animals in comparison to the sham CsA group (* *p* ≤ 0.05). The cFPI-induced loss of PV+ neurons was attenuated by the IL-1β neutralization (sham CsA, *n* = 4 sham IL-1β, *n* = 3; cFPI CsA, *n* = 7; cFPI IL-1β, *n* = 6; * *p* ≤ 0.05). (**D**) At 14 dpi, PV+ neurons loss was still detected in the GP (sham CsA, *n* = 3; sham IL-1β, *n* = 3; cFPI CsA, *n* = 8; cFPI IL-1β, *n* = 11; * *p* ≤ 0.05) of the cFPI CsA group compared with the sham CsA group; however, the effect of neutralizing IL-1 β did not reach significance. GP, globus pallidus; cFPI, central fluid percussion injury; dpi, days post-injury; CsA, inactive control antibody against cyclosporin A; IL-1β, interleukin-1 beta; PV, parvalbumin.

Cytokines released by activated microglia may contribute to degeneration of GABAergic neurons in the brain [30]. Most importantly, a loss of PV+ neurons have been described in animal models of Parkinson’s disease [31]. Therefore, we examined the number of PV+ interneurons in the GP at three different time points (2, 7, and 14 days) following TBI-induced by the cFPI model (Figure 2). At two dpi, there were no significant differences in the number of PV+ neurons between the sham- or brain-injured groups in the GP. However, at seven dpi, cFPI resulted in a 44% (sham CsA = 302 ± 44.5 and cFPI CsA = 133 ± 27; *p* < 0.05) decrease in the number of PV+ interneurons as compared to the control-treated sham group (sham CsA). IL-1β neutralizing treatment in brain-injured animals (the cFPI IL-1β group) resulted in normalized PV positive neurons numbers to those seen in the sham CsA group, and was significantly higher when compared to the cFPI CsA group (Figure 2A–C). At 14 dpi, the number of PV positive interneurons was still lower (*p* < 0.05) in the cFPI CsA group when compared with the sham CsA group, although not significantly different compared to the IL-1β neutralizing treatment groups (Figure 2D).

### 2.3. IL-1beta Neutralization Normalizes Increased Dopaminergic Innervation in the GP

**Figure 3:**
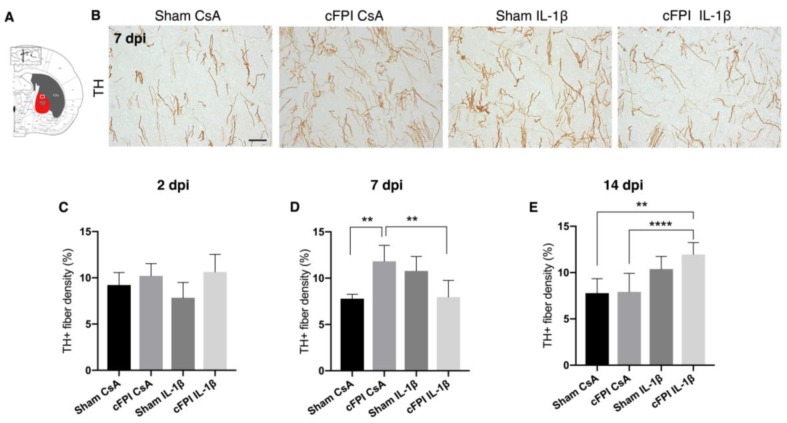
Effects of diffuse traumatic brain injury and neutralizing IL-1β antibody on tyrosine hydroxylase-positive axon density in the globus pallidus. (**A**) Schematic representation of a coronal brain section showing location of globus pallidus (GP) and GPe (framed area in (**A**)). (**B**) Representative images showing TH+ axons in the GP at seven dpi, scale bar 20 μm. (**C**) Compared with control-treated, sham-injured (sham CsA) animals at two dpi, there was no change in the density of TH+ axons (sham CsA, *n* = 4; sham IL-1β, *n* = 3; cFPI CsA, *n* = 6; cFPI IL-1β, *n* = 6) (**D**) At seven dpi, the density of TH+ axons were increased significantly in the control-treated, brain-injured (cFPI CsA) animals in comparison to sham CsA (sham CsA, *n* = 4; sham IL-1β, *n* = 3; cFPI CsA, *n* = 7; cFPI IL-1β, *n* = 6; ** *p* ≤ 0.01). TH+ axon density was attenuated in brain-injured animals treated with the IL-1B neutralizing antibody. (cFPI IL-1β; ** *p* ≤ 0.01). (**E**) At 14 dpi, no difference was observed in the density of TH+ axons in the cFPI CsA animals in comparison to sham CsA animals. The density of TH+ axons was significantly increased by IL-1β neutralization in the brain-injured mice as compared to Sham CsA and cFPI CsA groups (sham CsA, *n* = 3; sham IL-1β, *n* = 3; cFPI CsA, *n* = 8; cFPI IL-1β, *n* = 11); **** *p* ≤ 0.001). cFPI, central fluid percussion injury; dpi, days post-injury; CsA, inactive control antibody against cyclosporin A; IL-1β, interleukin 1 beta; TH, tyrosine hydroxylase; GP, globus pallidus.

The GP receives dopaminergic innervation by collaterals of nigrostriatal fibers that reach the striatum and TH+ axons establish a synaptic contact with neurons in the GP [32,33]. Given that TBI caused a significant decrease in the number of PV+ positive pallidal neurons, we next investigated the effects of neutralizing IL-1β antibody treatment on the dopaminergic innervation of the GP using immunohistochemistry to analyze the TH+ axon density in the external part of the GP (GPe) located close to the striatal boundary (Figure 3). At two dpi, the density of TH+ axons in GPe did not differ significantly among the groups. At seven dpi, the GPe of brain-injured control-treated animals (cFPI CsA) had increased TH+ axons density by 53% compared to the sham CsA group (Figure 3A–C). Neutralizing IL-1β antibody treatment in brain-injured animals, the cFPI IL-1β group, resulted in a decreased TH+ axon density when compared to the cFPI CsA group, showing values comparable to sham CsA group (Figure 3A,C). At 14 dpi, TH+ axon density in the cFPI CsA animals remained unchanged in comparison to the sham-treated animals. Here, there was a significant increase in the TH+ axon density in the cFPI IL-1β group when compared to the sham CsA and cFPI CsA groups (Figure 3D).

## 3. Discussion

In this study, we used the cFPI model of diffuse TBI in mice and observed microglia activation and neuronal changes in the GP that may contribute to the development of PD-like pathology. Furthermore, neutralization of the key pro-inflammatory cytokine IL-1β normalized the inflammatory response, and attenuated the loss of pallidal PV+ neurons and reduced TH+ axon density in the GP.

TBI is a markedly heterogeneous disease that causes persistent motor and sensory symptoms in patients, and it is closely associated with an increased risk of neurodegenerative diseases including PD [34,35,36,37]. Additionally, various preclinical studies have evaluated the relationship between experimental TBI and neurodegeneration. For instance, in rats subjected to controlled cortical impact, a model of focal TBI, degeneration of dopaminergic neurons was associated with decreased levels of tyrosine hydroxylase and dopamine transporter in the SN [38]. Moreover, rats subjected to the lateral fluid percussion TBI model showed a progressive cell loss of dopaminergic neurons in the SN and striatum and a higher vulnerability to paraquat, used to induce selective degeneration of dopaminergic neurons [21]. A progressive degeneration of dopaminergic neurons occurs along with microglia activation in TBI [21]. Likewise, mice exposed to closed head TBI showed a higher vulnerability to 6-hydroxydopamine injection [39]. Experimental TBI is also associated with accumulation of α-synuclein in activated microglial cells, resulting in injury to dopaminergic neurons [20].

The activation of microglia in the GP has previously been recognized in other neurodegenerative diseases [40]. In this study, the cFPI model of diffuse TBI was used to produce wide-spread axonal injury without focal hemorrhagic lesions and triggered a microglia response in the GP similar to that observed in the white matter [12,41]. IL-1β contributes to microglia activation and acts as a strong proliferative stimulus for microglia in injured brain tissues, probably through P2X7 purinergic receptors [42,43]. However, the detailed mechanisms involved in TBI-induced microglia activation in GP remain to be investigated. Our findings of attenuated microglia activation in the GP by IL-1β neutralization support the hypothesis that IL-1β is a crucial stimulus for altered microglial response [4,5,12]. These results also indicate that the antibody penetrated into the injured brain in sufficient concentrations needed to neutralize IL-1β following cFPI [41]. Indeed, we have previously shown that intraperitoneal administration of the IL-1β neutralizing antibody at 30 min post-injury resulted in high penetration of the antibody into the injured brain [44,45]. Surprisingly, IL-1β neutralizing antibody treatment resulted in an increase in the number of Iba1+ microglia cells in the GP at 14 dpi. This could be due to a possible aggregation of IL-1β by the neutralizing antibody that potentiates its action, which may have an opposite effect on microglia. However, we did not directly measure the antibody concentration in the GP in the present study.

Mild TBI results in loss of GABAergic interneurons and inhibitory synaptic transmission in the hippocampus [46]. We observed a significant PV+ neuronal loss in the GP of brain-injured animals. Consistently, similar findings have been reported in rodent and primate models of PD [31]. This PV+ interneurons loss in the GP was attenuated by the treatment with an IL-1β receptor antagonist in a another model of TBI, the lateral fluid percussion model in rats [47]. Our present results are in line with a previous study showing that enhanced levels of IL-1β in the hippocampus, secreted by activated microglia, resulted in degeneration of GABAergic interneurons [30]. Interestingly, the loss of PV+ interneurons observed in this study was associated with increased number of microglia cells in GP. Microglia are well known to induce neuronal death due to the release of pro-inflammatory cytokines in neurodegenerative diseases, including PD. Increased levels of TBI-induced pro-inflammatory cytokines released by activated microglia can cause glutamate-mediated synaptic hyperexcitability and neurotoxicity [48,49,50]. Therefore, we cannot exclude the possibility that IL-1β neutralization has a direct effect on hyperexcitability at the synaptic level, caused by selective vulnerability of GABAergic interneurons in the GP of brain-injured animals. On the other hand, IL-1β-mediated glutamate toxicity is also involved in oligodendrocyte death [8]. In support, we recently showed that TBI-induced oligodendrocyte cell loss in the corpus callosum was attenuated by IL-1β neutralization [12]. In addition to glutamate excitoxicity, IL-1β-mediated toxicity was associated with caspase activation, release of free radicals and pro-matrix metallopeptidase-9 [9].

Finally, our study provides the first evidence that TBI induces a significant increase in the percentage of dopaminergic (DA) axons in the GP, in particular in its dorsal regions close to the striatal border. These results are consistent with positron emission tomography (PET) studies in PD patients showing that the pallidal DA innervation increases in the early stages of the disease [51,52]. Similar results were also reported in macaques treated with the neurotoxin 1-methyl-4-phenyl-1,2,3,6-tetrahydropyridine (MPTP) [53]. In support, our findings suggest that increased DA pallidal projection could be a compensatory mechanism to lower uncontrolled GP activity due to loss of parvalbumin-positive neurons following TBI [53]. Importantly, IL-1β neutralization normalized the DA axon density in the GP in addition to its effect on survival of pallidal PV+ neurons. Although we did not determine the endogenous levels of IL-1β in the GP and SN following injury and treatment, as one of the limitations of the study, our results suggest that chronic exacerbation of IL-1β affects DA neurotransmission and innervation in the basal ganglia, in particular the GP. It is noteworthy that chronic expression of IL-1β and microglia activation may impair dopaminergic neurotransmission and lead to DA cell death in the SN [54,55].

Modulation of the inflammatory cascades is emerging as a promising treatment target in clinical TBI. Using an interleukin-1 receptor antagonist (IL1ra), important changes to the chemokine and cytokine profile, as measured by cerebral microdialysis, were observed in severe human TBI [56,57]. Furthermore, tumor necrosis factor alpha (TNF-α) is another important mediator of the inflammatory response in TBI, reviewed in [58]. To date, TNF-α has been evaluated mainly in experimental TBI models; however, several TNF-α targeting drugs, including e.g., infliximab and others have been approved for clinical use in autoimmune inflammatory diseases and evaluation in clinical TBI may be expected in the near future. The inflammasome is a crucial contributor to the inflammatory response, and IL-18 and other inflammasome associated proteins are increased in serum, cerebrospinal fluid and extracellular fluid in response to experimental and human TBI [59,60,61]. Furthermore, treatment with the naturally occurring inhibitor IL-18-binding protein (IL-18BP) improved recovery following experimental TBI [62]. Arguably, IL-18 may be an additional contributor to the microglia activation shown here. However, blood samples were not obtained in the present study. A time course of IL-1β and IL-18 in blood should be addressed to evaluate the effects of the study antibody in future studies.

Since the IL-1β neutralization antibody altered the time course of the TBI-induced changes evaluated in the present report, further studies are needed to understand the effects of neuroinflammation on the development of PD-like pathology. In addition, we only evaluated the changes up to 14 days post-injury and additional survival time points should be evaluated in follow-up studies.

Regardless of these limitations, this study supports the hypothesis that the neuroinflammatory response associated with TBI is linked to the mechanisms resulting in a post-injury risk increase for neurodegenerative diseases such as PD. Increased IL-1β levels after TBI may elicit basal ganglia dysfunction and parkinsonism-related outcomes that can be reversed by IL-1β neutralization.

## 4. Materials and Methods

### 4.1. Animals and Central Fluid Percussion Model

Adult male mice C57BL/6 mice (pre-injury weight 25 ± 1.7 g; Taconic, Silkeborg, Denmark), were housed with free access to food and water for a minimum of seven days prior to surgery. The study followed the regulations of the Swedish Animal Welfare Agency (Uppsala, Sweden) and experiments were approved by the Ethical Committee at Uppsala County (C46/15 Date: 24-05-2015).

The surgical procedure for central (midline) fluid percussion injury (cFPI; *n* = 78) has been described in detail previously [12], and the present study is a follow-up study based on this report. Briefly, anesthesia was induced by placing the mouse in a ventilated Plexiglas container with 4% isoflurane in air, and then maintained by isoflurane 1.2% and N_2_O/O_2_ 70/30% delivered through nosecone delivering. A 3.0 mm diameter craniotomy was created over the midline, keeping the dura mater and the sagittal sinus intact, and a plastic cap was attached and sealed over the craniotomy. Then, the cap was filled with isotonic saline at room temperature and attached to the Luer-Lock on the fluid percussion device (VCU Biomedical Engineering Facility, Richmond, VA, USA). Brain injury was induced by releasing the fluid percussion pendulum striking the saline-filled cylinder, creating a pressure wave subsequently transmitted into the cranial cavity. The injury-induced apnea and immediate post-injury seizures were recorded.

Sham-injured animals (*n* = 38) were subjected to anesthesia and surgery, although the pendulum was not released. After surgery, the cap was removed, the bone flap replaced and the skin sutured using resorbable sutures. The cFPI-related mortality was 13% [12], and the tissue from five animals could not be analyzed due to technical issues (inadequate perfusion or tissue sectioning). In total, 91 animals were included.

### 4.2. IL-1β Neutralizing Antibody Administration

The IL-1β neutralizing antibody (IL-1β; 300 μg/dose) or control anti-cyclosporin A mlgG2a (CsA; 500 μg/dose), kindly provided by Novartis, Inc., Basel, Switzerland, was administered intraperitoneal (i.p.) 30 min after sham injury or cFPI for all evaluated groups. The 14 dpi group received a second i.p. injection with the same dose at seven dpi.

### 4.3. Tissue Preparation

The mice were sacrificed by i.p.-injection of sodium pentobarbital (Pentobarbitalnatrium 400 mg/mL, VET ATL, Apoteket, Uppsala, Sweden; 200 mg/kg) at 2, 7 or 14 dpi and subsequently transcardially perfused using 4% formaldehyde (formaldehyde 4% Fosfatbuffrad, HistoLab Products AB, Gothenburg, Sweden). The brains were post-fixed overnight at 4 °C, and placed in 30% sucrose solution for 72 h until snap frozen in isopentane and stored at −70 °C until sectioned.

Brains were cut in 20 μm thick coronal sections and kept in cryoprotectant buffer (30% glycerin, 30% ethylene glycol, and 40% 1× phosphate buffered saline [PBS]) at −20 °C until further tissue processing.

### 4.4. Immunohistochemistry

Brain coronal sections were washed three times in phosphate buffered saline (PBS) and quenched with 3% H_2_O_2_ for 20 min. Afterwards, sections were blocked with 5% normal donkey serum (NDS) in PBS supplemented with 0.25% Triton X-100 (PBS-T) for one hour. Primary antibodies were incubated overnight at room temperature or at 4 °C in 3% NDS PBS-T. The following primary antibodies were used: TH (rabbit cat# AB152, EMD Millipore, Temecula, CA, USA, 1:1000), Parvalbumin (goat cat# PVG213, Swant, Marly, Fribourg, Switzerland, 1:3000), Iba1 (rabbit cat# 019-19741, Fujifilm Wako Pure Chemical Corporation, Osaka, Japan, 1:1000). After incubation with primary antibody, sections were incubated with corresponding biotinylated secondary antibodies (Jackson Immunoresearch, Baltimore, MA, USA, 1:400), in 3% NDS PBS-T, at room temperature for 90 min, and the signal was enhancement by using Vectorstain ABC Elite kit (Vector Laboratories, Burlingame, CA, USA). Staining was revealed using chromogen 3,3-diaminobenzidine-tetrahydrochloride (Dabsafe, Saveen Werner AB, Limhamn, Sweden) and 3% H_2_O_2_. Sections were dehydrated in consecutive higher concentrations of ethanol, followed by xylene and mounted using Pertex (Histolab AB, Gothenburg, Sweden).

### 4.5. Image Processing and Cell Quantification

3,3′-Diaminobenzidine (DAB) stained sections were analyzed using Olympus BX51 light microscope (Olympus, Tokyo, Japan), and cells were counted using Olympus CellSens Dimension 1.5 digital imaging software (Olympus, Tokyo, Japan). Figures were composed using Photoshop CS5 software (Adobe, Mountain View, CA, USA).

To quantify the number of DAB-stained Iba1 positive microglia cells, an optical field of 0.013 mm^2^ in the globus pallidus from each section was taken at 20×. Nuclei of the Iba-1 microglia cells were quantified.

To analyze TH+ pallidal axon density, one high-resolution picture of the external part of the GP (GPe) located close to the striatal boundary per brain was obtained at magnification 20×, using bright field Olympus BX51 microscope. A ROI size of 0.084 mm^2^ for bright field images was used. The area covered by TH-positive axons was analyzed using the NIH ImageJ area measurement tool where pictures were subjected to threshold processing to produce a binary image. The area is expressed as percentage of the total area.

### 4.6. Statistics

Graphs and statistical analysis were made with GraphPad Prism v.7.0c (GraphPad Software, La Jolla, CA, USA). Normality of data distribution was analyzed by Shapiro-Wilk normality test. One-way ANOVA was used followed by Tukey multiple comparison test. In graphs, data is presented as mean and standard error of the mean. Significance was set at *p* < 0.05.
